# Large Left Ventricular Thrombus in a Patient with Systemic and Venous Thromboembolism Secondary to Protein C and S Deficiency

**DOI:** 10.1155/2017/7576801

**Published:** 2017-01-04

**Authors:** Mohit Pahuja, Bujji Ainapurapu, Aiden Abidov

**Affiliations:** ^1^Department of Medicine, St. Joseph's Hospital and Medical Center, Phoenix, AZ, USA; ^2^Department of Medicine, Banner University Medical Center, Tucson, AZ, USA; ^3^Section of Cardiology, John D. Dingell VA Medical Center, Detroit, MI, USA; ^4^Department of Medicine, Wayne State University, Detroit, MI, USA

## Abstract

58-year-old Hispanic female presented with an altered mental status. A CT scan of the head demonstrated multiple scattered infarcts and a large right temporal lobe infarct. We also diagnosed the patient with right popliteal and femoral vein thrombosis, bilateral pulmonary embolism, and a transient right radial artery occlusion. Her 12-lead EKG showed lateral ST elevation. Emergent coronary angiogram revealed normal coronaries. Echocardiogram demonstrated a large mobile mass attached to the anterolateral free wall with overall normal contractility of the left ventricle. The patient underwent surgical embolectomy to prevent further systemic embolization. Coagulability workup returned positive for protein C and S deficiency. The patient did well after surgery. Following her surgery, we initiated chronic oral anticoagulation. The presentation with intracardiac thrombus in a normal heart should raise a concern of a probable thrombophilia.

## 1. Introduction

Protein C and S deficiency is an uncommon type of thrombophilic genetic syndromes. Deficiency of both protein C and S may result in increased risk of venous and arterial thrombosis [1]. Cases of cardiac/nonvascular thrombosis appear to be exceptionally rare [2, 3].

We present a case of a patient admitted with a right temporal lobe infarct along with bilateral segmental pulmonary embolism and deep vein thrombosis. Further workup revealed a large mobile left ventricular thrombus. Hematological workup revealed findings consistent with combined protein C and S deficiency. The patient underwent an urgent surgical cardiac thrombectomy and was subsequently treated with anticoagulation therapy.

## 2. Case Presentation 

A 58-year-old Hispanic female with a history of hypertension and hyperlipidemia was brought to the emergency department for an altered mental status. On admission, the patient was hemodynamically stable. She had a nonfocal initial neurologic exam. Her laboratory findings were consistent with hypoxemia, acidosis, and mildly elevated leukocytosis. A 12-lead ECG revealed sinus tachycardia with lateral ST segment elevation; due to these findings, the patient underwent emergent cardiac catheterization, which revealed normal coronaries.

A noncontrast CT scan of the head demonstrated a right subacute temporal lobe infarct and multiple scattered infarcts in all vascular territories raising the possibility of embolic phenomenon. Further clinical course was complicated by right popliteal and superficial femoral vein thrombosis and bilateral segmental pulmonary embolism. The patient also had a transient right radial artery occlusion.

**Figure 1:**
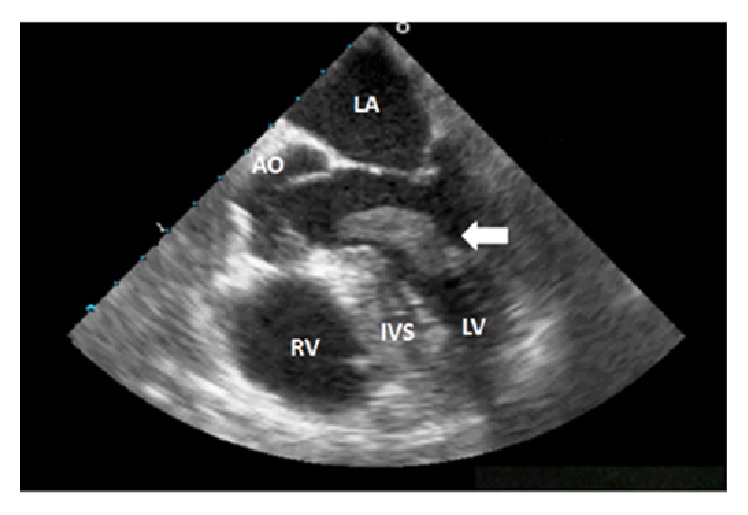
Large mass in the left ventricle (white arrow) extending into the left ventricular outflow tract. AO = aortic root; IVS = interventricular septum; LA = left atrium; LV = left ventricle; RV= right ventricle.

Her initial contrast-enhanced transthoracic echocardiography (performed after the cardiac catheterization) and subsequent transesophageal echocardiography (TEE) revealed a large left ventricular mass with a wide base, attached to the anterolateral free wall. The mass had poorly defined edges and several mobile components. There were no other significant structural and functional cardiac abnormalities noted. Detailed TEE analysis (Figure 1, Supplementary video in Supplementary Material available online at https://doi.org/10.1155/2017/7576801) confirmed a presence of a 42 × 10 mm thrombus arising from the lateral wall and extending into the left ventricular outflow tract.

Evidence of multiple arterial and venous clots in addition to the large cardiac thrombus raised the suspicion for inherited thrombophilia. The coagulability workup was performed and came back positive for protein C and S deficiency, with activity of 26% (normal range: 84–171) and 18% (normal range: 54–132% for females) for proteins C and S, respectively. At that time, the patient had normal liver function and vitamin K level and was not on any vitamin K antagonist therapy. Renal function was normal and the patient did not have any evidence of disseminated intravascular coagulation.

**Figure 2:**
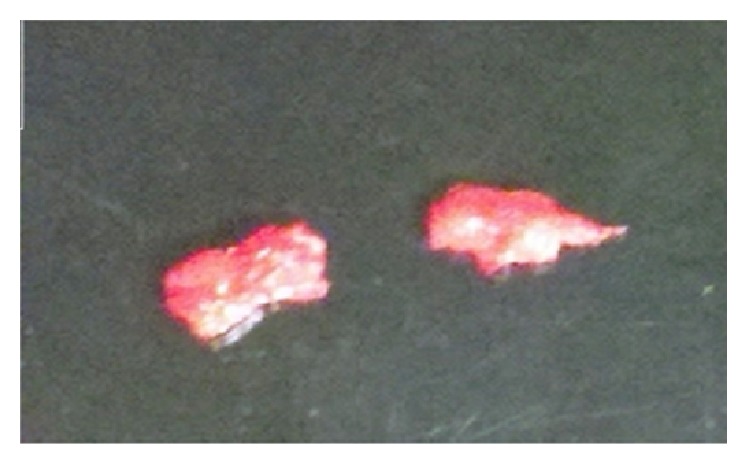
Intraoperative photograph demonstrates a large elongated soft pink-tan thrombus excised from the left ventricle. Due to its size, the thrombus was extracted in 2 pieces.

The patient underwent a surgical cardiac thrombectomy to prevent further systemic embolization. An elongated mass attached to the lateral wall and connected to anterior mitral leaflet was removed (Figure 2). No valvular changes or lateral wall regional wall motion abnormalities were observed. Pathology confirmed an organizing arterial thrombus without evidence of infection. The patient did well after surgery and was treated with oral anticoagulation (Warfarin, bridged with intravenous Heparin) and discharged to skilled nursing facility with no further cardiac or vascular complications.

## 3. Discussion

Protein C and protein S are vitamin K-dependent plasma proteins. Activated protein C in the presence of protein S inhibits activated coagulation factors V and VIII. Protein S acts as cofactor for the protein C system. Both protein C and S deficiency are autosomal dominant genetic disorders, usually manifesting as venous thromboembolism with deep and superficial vein thrombosis, pulmonary embolism, or both. Rarely, protein C and S deficiency is associated with an arterial thrombosis as well as nonhemorrhagic stroke [4–6]. In a cohort study containing three cohorts of families with a hereditary deficiencies of protein C, protein S, and antithrombin III, compared with nondeficient family members, subjects with protein S or protein C deficiency but not antithrombin III deficiency had a higher risk for arterial thromboembolism before 55 years of age that was independent of their history of prior venous thromboembolism [4]. In the ARIC (Atherosclerosis Risk in Communities) study, ischemic stroke had a negative but nonsignificant association with protein C levels after adjustment for all other risk factors [7].

Intracardiac thrombus formation can occur in patients with protein C and S deficiency but is relatively rare in comparison to venous and arterial thrombosis. Most of the cases reported association with other risk factors such as high altitude, left ventricular dysfunction, or dilated cardiomyopathy [2, 3, 8–10]. Possible mechanisms include increased hyperviscosity due to dehydration, stasis, and decreased intracardiac blood flow [11–13]. It is unusual to develop intraventricular thrombus with a normal contractility in absence of cardiomyopathy with either global or regional left ventricular dysfunction. In this regard, Özkutlu et al. demonstrated that three out of eleven patients with intracardiac thrombus and otherwise normal heart had protein C deficiency [14]. Our patient did not have any cardiomyopathy or left ventricular dysfunction. We report an extremely rare case of such an extensive mobile cardiac thrombus with multiple arterial and venous embolic events with combined protein C and S deficiency. We propose that patients presenting with large intraventricular thrombus should be checked for protein C and S deficiency especially when associated with venous and arterial thrombosis.

Currently there are no guidelines available for the management of intracardiac thrombus. The majority of the patients are placed on anticoagulation therapy. Lee et al. proposed that, in comparison to patients with nonpedunculated thrombi, patients with mural pedunculated thrombi are at increased risk for both systemic embolization and recurrent embolism despite anticoagulation therapy. Risk of systemic embolization was higher in patients on anticoagulant therapy compared to surgical therapy (17.7% versus 0%) [15]. Surgical thrombectomy appears to be clinically effective in cases with mobile pedunculated thrombi [16–20]. In our case, due to multiple embolic phenomena and increased risk of new embolization in this patient with thrombophilia, we decided to proceed with surgical thrombectomy with an excellent clinical outcome.

## 4. Conclusion

We conclude that protein C and S deficiency should be considered in the differential diagnosis in patients presenting with intracardiac and systemic thrombosis. Advanced cardiovascular imaging may prove useful in determining the need for surgical thrombectomy. Systemic anticoagulation therapy is the most appropriate therapeutic option in patients with intracardiac and systemic thrombosis.

## Supplementary Material

TEE video shows a large mobile mass in the left ventricle extending into the left ventricular outflow tract.
